# Supplementary material for: Acanthaster planci Inhibits PCSK9 and Lowers Cholesterol Levels in Rats
Source: Molecules. 2021 Aug 23;26(16):5094. doi: 10.3390/molecules26165094 (PMC8398678; doi:10.3390/molecules26165094)
Supplement: Supplementary file 1 [file molecules-26-05094-s001.zip › molecules-1296797-supplementary.pdf]

A

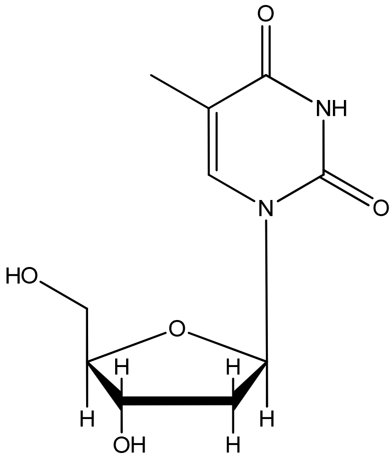
O
NH
N
O
HO
O
H
H
H
H
OH
H

B

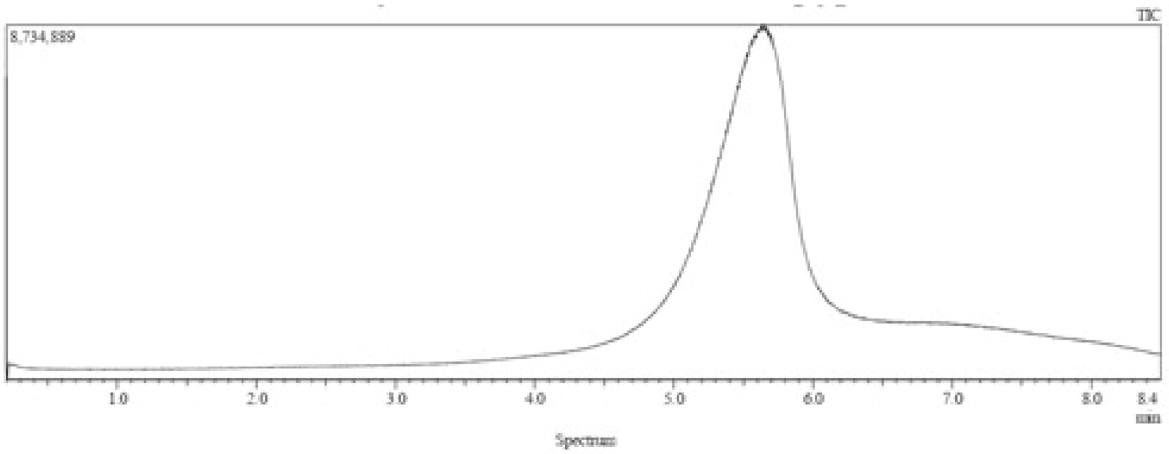
8,734,889
TIC
1.0
2.0
3.0
4.0
5.0
6.0
7.0
8.0
8.4
min
Spectrum

C

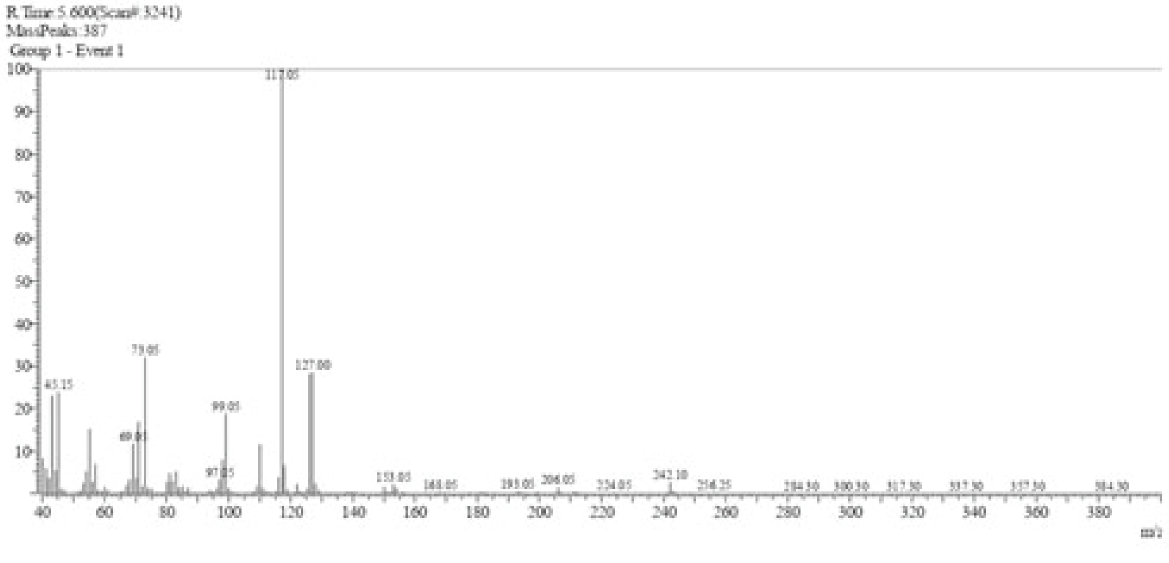
R.Time:5.600(Scan#:3241)
MassPeaks:387
Group 1 - Event 1
117.05
73.05
127.00
99.05
40
60
80
100
120
140
160
180
200
220
240
260
280
300
320
340
360
380
m/z

D

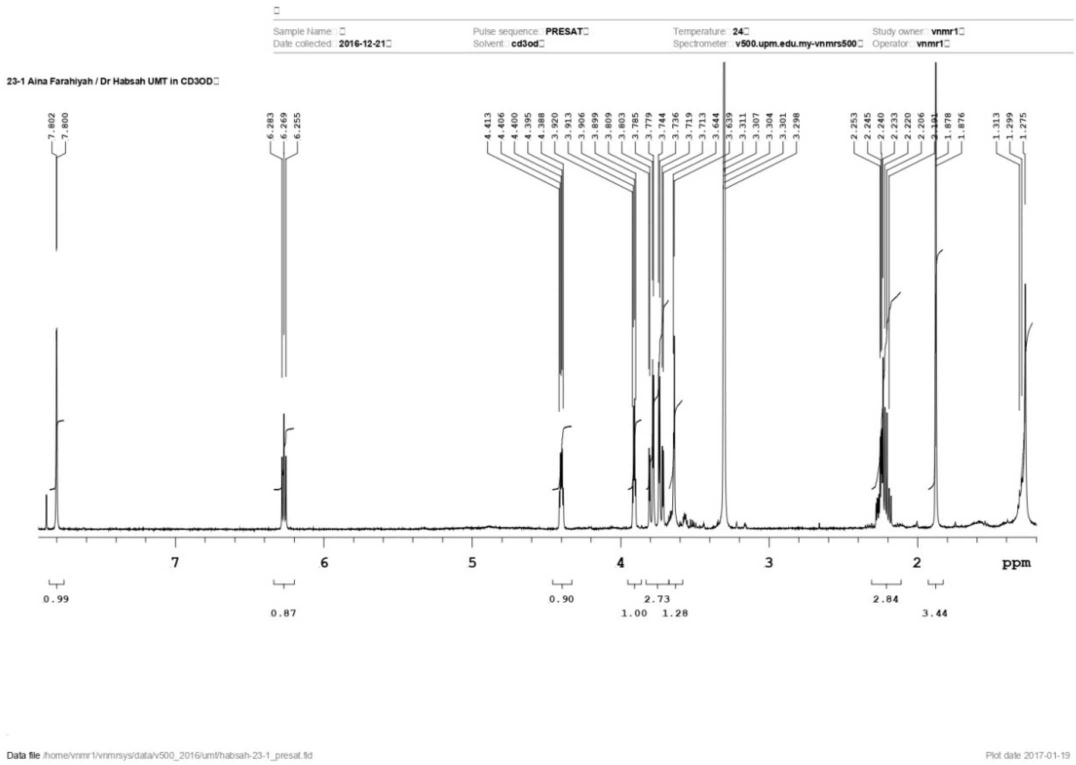
Sample Name: Date collected: 2016-12-21
Pulse sequence: PRESAT Solvent: cd3od
Temperature: 24 Spectrometer: v500.upm.edu.my-vnmrs500
Study owner: vnmr1 Operator: vnmr1
23-1 Aina Farahiyah / Dr Habsah UMT in CD3OD
7.802 7.800 6.283 6.269 6.255 4.413 4.406 4.400 4.395 4.388 3.920 3.913 3.906 3.899 3.809 3.803 3.785 3.779 3.744 3.736 3.719 3.713 3.644 3.639 3.311 3.307 3.304 3.301 3.298 2.253 2.245 2.240 2.233 2.220 2.206 2.191 1.878 1.876 1.313 1.299 1.275
7 6 5 4 3 2 ppm
0.99 0.87 0.90 1.00 2.73 1.28 2.84 3.44
Data file /home/vnmr1/vnmrsys/data/v500_2016/umt/habsah-23-1_presat.fid
Plot date 2017-01-19

E

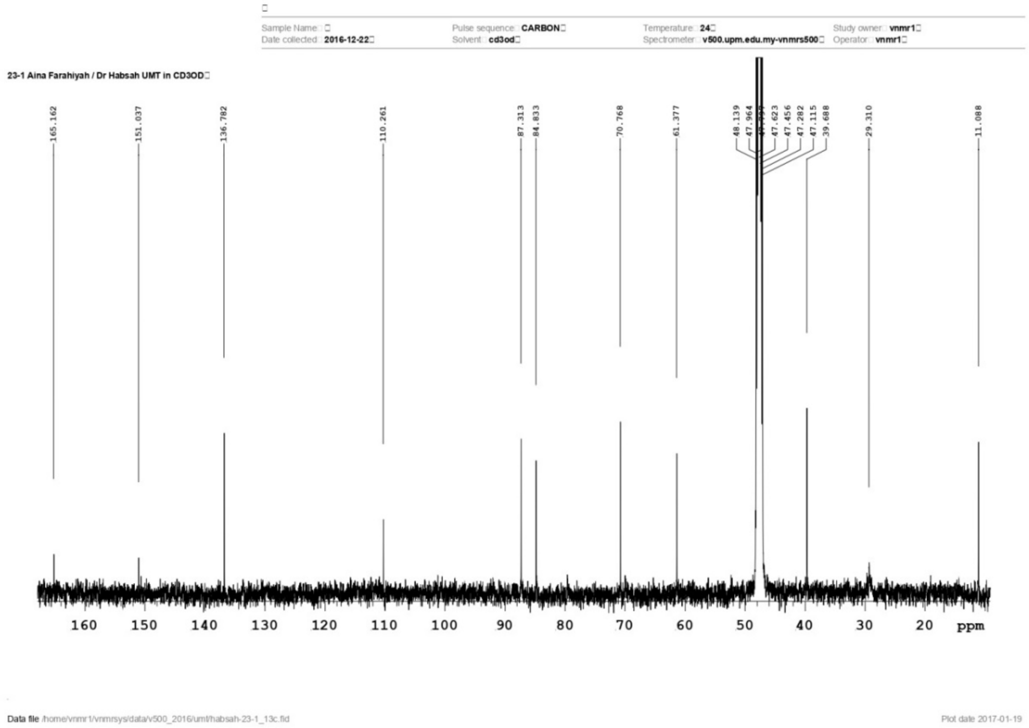
Sample Name: Date collected: 2016-12-22
Pulse sequence: CARBON Solvent: cd3od
Temperature: 24 Spectrometer: v500.upm.edu.my-vnmrs500
Study owner: vnmr1 Operator: vnmr1
23-1 Aina Farahiyah / Dr Habsah UMT in CD3OD
165.162 151.037 136.782 110.261 87.313 84.833 70.768 61.377 48.139 47.964 47.623 47.456 47.282 47.115 39.688 29.310 11.088
160 150 140 130 120 110 100 90 80 70 60 50 40 30 20 ppm
Data file /home/vnmr1/vnmrsys/data/v500_2016/umt/habsah-23-1_13c.fid
Plot date 2017-01-19

Supplementary figure. Structure elucidation of a compound isolated from EF2 *Acanthaster planci* (A) The chemical structure of thymidine; (B) Total ion chromatogram; (C) Mass Chromatogram; (D) ^{1}H NMR spectrum; (E) ^{13}C NMR spectrum.
